# 4-Methyl-2,3-dihydro-1*H*-1,5-benzodiazepin-2-one monohydrate

**DOI:** 10.1107/S1600536810017885

**Published:** 2010-05-22

**Authors:** Asmaa Saber, Hafid Zouihri, El Mokhtar Essassi, Seik Weng Ng

**Affiliations:** a Laboratoire de Chimie Organique Hétérocyclique, Pôle de Compétences Pharmacochimie, Université Mohammed V-Agdal, BP 1014 Avenue Ibn Batout, Rabat, Morocco; b CNRST Division UATRS, Angle Allal Fassi/FAR, BP 8027 Hay Riad, Rabat, Morocco; c Department of Chemistry, University of Malaya, 50603 Kuala Lumpur, Malaysia

## Abstract

The seven-membered fused-ring in the title compound, C_10_H_10_N_2_O·H_2_O, adopts a boat conformation (with the two phenyl­ene C atoms representing the stern and the methyl­ene C atom the prow). In the crystal, two benzodiazepinone mol­ecules are linked about a center of inversion by diazepine–carbonyl N—H⋯O hydrogen bonds. The dimers are further linked by water–diazepine O—H⋯N hydrogen bonds, forming a linear chain.

## Related literature

For background to the synthesis and biological activity of benzodiazepines, see: Ahabchane *et al.* (1999 ▶). For the microwave-assisted synthesis, see: Koizumi (2006 ▶). For a related structure, see: Saber *et al.* (2010 ▶).
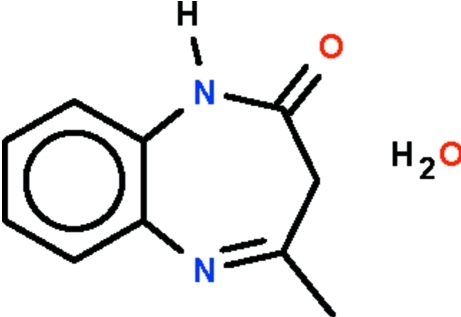


## Experimental

### 

#### Crystal data

C_10_H_10_N_2_O·H_2_O
*M* *_r_* = 192.22
Triclinic, 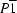

*a* = 4.9013 (1) Å
*b* = 7.3148 (1) Å
*c* = 13.5688 (2) Å
α = 85.375 (1)°
β = 83.959 (1)°
γ = 83.807 (1)°
*V* = 479.76 (1) Å^3^
*Z* = 2
Mo *K*α radiation
μ = 0.10 mm^−1^
*T* = 100 K
0.43 × 0.27 × 0.25 mm

#### Data collection

Bruker X8 APEXII diffractometer
14168 measured reflections
2778 independent reflections
2417 reflections with *I* > 2σ(*I*)
*R*_int_ = 0.025

#### Refinement

*R*[*F* ^2^ > 2σ(*F* ^2^)] = 0.038
*wR*(*F* ^2^) = 0.115
*S* = 1.01
2778 reflections
144 parameters
6 restraints
H atoms treated by a mixture of independent and constrained refinement
Δρ_max_ = 0.33 e Å^−3^
Δρ_min_ = −0.23 e Å^−3^

### 

Data collection: *APEX2* (Bruker, 2008 ▶); cell refinement: *SAINT* (Bruker, 2008 ▶); data reduction: *SAINT*; program(s) used to solve structure: *SHELXS97* (Sheldrick, 2008 ▶); program(s) used to refine structure: *SHELXL97* (Sheldrick, 2008 ▶); molecular graphics: *X-SEED* (Barbour, 2001 ▶); software used to prepare material for publication: *publCIF* (Westrip, 2010 ▶).

## Supplementary Material

Crystal structure: contains datablocks global, I. DOI: 10.1107/S1600536810017885/nc2183sup1.cif

Structure factors: contains datablocks I. DOI: 10.1107/S1600536810017885/nc2183Isup2.hkl

Additional supplementary materials:  crystallographic information; 3D view; checkCIF report

## Figures and Tables

**Table 1:** Hydrogen-bond geometry (Å, °)

*D*—H⋯*A*	*D*—H	H⋯*A*	*D*⋯*A*	*D*—H⋯*A*
N2—H2⋯O1^i^	0.87 (1)	2.05 (1)	2.909 (1)	171 (1)
O1w—H11⋯N1	0.83 (1)	2.12 (1)	2.945 (1)	170 (2)
O1w—H13⋯O1w^ii^	0.84 (1)	1.98 (1)	2.803 (2)	167 (5)

Symmetry codes: (i) 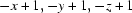; (ii) 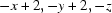.

**Fig. 1.**
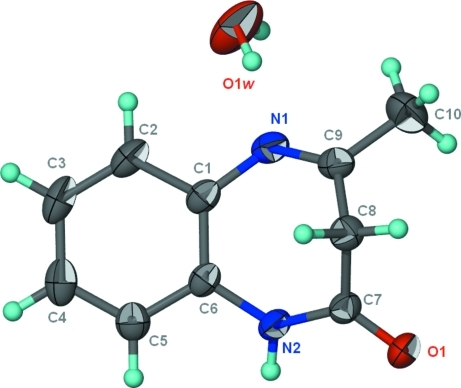
Thermal ellipsoid plot (Barbour, 2001) of the molecule of C10H10N2O.H2O at the 70% probability level; hydrogen atoms are drawn as spheres of arbitrary radius. The disorder in one of the two the hydrogen atoms of the water molecule is not shown.

## supplementary crystallographic information

### Comment

The compound is belongs to the class of benzodiazepine drugs; the compound is synthesized by condensing *o*-phenylenediamine with ethyl acetoacetate, two readily-available commerically chemicals. The chemical background of this class of precusor compounds is presented in an earlier report (Ahabchane *et al.*, 1999). A more recent study reports the microwave-assisted synthesis of the title compound (Koizumi, 2006), which is presumably anhydrous. However, the compound crystallizes as a monohydrate (Scheme I, Fig. 1), as shown from the crystal structure analysis.

One of the two hydrogen atoms of the water molecule is disordered over two positions in a 1:1 ratio. That hydrogen atom near the center-of-inversion is hydrogen bonded to the inversion-related oxygen atom. As the benzodiazepinone molecule is N–H···O hydrogen bonded into a dimer, the water molecule then links adjacent dimers into a linear chain (Table 1).

### Experimental

*o*-Phenylenediamine (1.0 g, 9 mmol) and ethyl acetoacetate (1.2 ml, 9 mmol) were heated in xylene (10 ml) for 1 hour. The mixture was set aside for the growth of colorless crystals of 4-methyl-2,3-dihydro-1*H*-1,5-benzodiazepin-2-one; yield 90%. When the heating time is lengthened to 6 hours, the product is *N*-isopropenyl 1,3-benzimidazol-2-one; details are given in another report (Saber *et al.*, 2010).

### Refinement

Carbon-bound H-atoms were placed in calculated positions (C—H 0.95–0.98 Å) and were included in the refinement in the riding model approximation, with *U*(H) set to 1.2–1.5*U*_eq_(C).

One of the two hydrogen atoms of the water molecule is disordered over two positions. The hydrogen atom near the center-of-inversion should have only half occupancy; this is linked to the inversion-related water molecule. The O–H distances were restrained to 0.84±0.01 Å and the H···H distances to 1.37±0.01 Å; the isotropic temperature factors of the hydrogen atoms were freely refined.

The amino H-atom was located in a difference Fourier map, and was refined with a distance restraint of N–H 0.86±0.01 Å; its temperature factor was also freely refined.

### Crystal data

**Table:**

C_10_H_10_N_2_O·H_2_O	*Z* = 2
*M**_r_* = 192.22	*F*(000) = 204
Triclinic, *P*1	*D*_x_ = 1.331 Mg m^−^^3^
Hall symbol: -P 1	Mo *K*α radiation, λ = 0.71073 Å
*a* = 4.9013 (1) Å	Cell parameters from 7100 reflections
*b* = 7.3148 (1) Å	θ = 2.8–37.4°
*c* = 13.5688 (2) Å	µ = 0.10 mm^−^^1^
α = 85.375 (1)°	*T* = 100 K
β = 83.959 (1)°	Block, colorless
γ = 83.807 (1)°	0.43 × 0.27 × 0.25 mm
*V* = 479.76 (1) Å^3^	

### Data collection

**Table:**

Bruker X8 APEXII diffractometer	2417 reflections with *I* > 2σ(*I*)
Radiation source: fine-focus sealed tube	*R*_int_ = 0.025
graphite	θ_max_ = 30.0°, θ_min_ = 2.8°
φ and ω scans	*h* = −6→6
14168 measured reflections	*k* = −10→10
2778 independent reflections	*l* = −19→19

### Refinement

**Table:**

Refinement on *F*^2^	Primary atom site location: structure-invariant direct methods
Least-squares matrix: full	Secondary atom site location: difference Fourier map
*R*[*F*^2^ > 2σ(*F*^2^)] = 0.038	Hydrogen site location: inferred from neighbouring sites
*wR*(*F*^2^) = 0.115	H atoms treated by a mixture of independent and constrained refinement
*S* = 1.01	*w* = 1/[σ^2^(*F*_o_^2^) + (0.0699*P*)^2^ + 0.0787*P*] where *P* = (*F*_o_^2^ + 2*F*_c_^2^)/3
2778 reflections	(Δ/σ)_max_ = 0.001
144 parameters	Δρ_max_ = 0.33 e Å^−^^3^
6 restraints	Δρ_min_ = −0.23 e Å^−^^3^

### Fractional atomic coordinates and isotropic or equivalent isotropic displacement parameters (Å^2^)

**Table:**

	*x*	*y*	*z*	*U*_iso_*/*U*_eq_	Occ. (<1)
O1	0.50754 (15)	0.32480 (10)	0.41041 (5)	0.03282 (18)	
O1W	0.7622 (2)	0.90681 (16)	0.01525 (8)	0.0615 (3)	
H11	0.781 (4)	0.8214 (18)	0.0590 (11)	0.067 (5)*	
H12	0.597 (3)	0.941 (5)	0.009 (3)	0.082 (13)*	0.50
H13	0.891 (6)	0.975 (4)	0.012 (3)	0.094 (15)*	0.50
N1	0.89510 (16)	0.62527 (11)	0.17532 (6)	0.02795 (18)	
N2	0.74476 (15)	0.57576 (10)	0.39494 (5)	0.02466 (17)	
H2	0.654 (3)	0.6122 (18)	0.4498 (8)	0.037 (3)*	
C1	1.02194 (17)	0.70536 (12)	0.24757 (7)	0.02450 (18)	
C2	1.21868 (19)	0.82648 (13)	0.21134 (8)	0.0313 (2)	
H2A	1.2708	0.8399	0.1419	0.038*	
C3	1.3380 (2)	0.92630 (13)	0.27404 (9)	0.0348 (2)	
H3	1.4753	1.0043	0.2481	0.042*	
C4	1.2567 (2)	0.91253 (13)	0.37537 (9)	0.0335 (2)	
H4	1.3346	0.9837	0.4187	0.040*	
C5	1.06207 (19)	0.79500 (13)	0.41311 (7)	0.02855 (19)	
H5	1.0057	0.7871	0.4824	0.034*	
C6	0.94708 (16)	0.68755 (11)	0.35054 (6)	0.02275 (18)	
C7	0.69461 (17)	0.41042 (12)	0.36746 (6)	0.02391 (18)	
C8	0.88337 (18)	0.33830 (12)	0.28103 (7)	0.02627 (19)	
H81	0.8500	0.2101	0.2715	0.032*	
H82	1.0783	0.3389	0.2940	0.032*	
C9	0.82545 (18)	0.46102 (13)	0.18931 (7)	0.02720 (19)	
C10	0.6764 (3)	0.38563 (18)	0.11355 (9)	0.0449 (3)	
H10A	0.7754	0.2688	0.0934	0.067*	
H10B	0.6667	0.4740	0.0555	0.067*	
H10C	0.4893	0.3646	0.1421	0.067*	

### Atomic displacement parameters (Å^2^)

**Table:**

	*U*^11^	*U*^22^	*U*^33^	*U*^12^	*U*^13^	*U*^23^
O1	0.0366 (4)	0.0335 (4)	0.0295 (3)	−0.0160 (3)	0.0020 (3)	0.0010 (3)
O1W	0.0490 (6)	0.0668 (7)	0.0649 (6)	−0.0157 (5)	−0.0123 (5)	0.0400 (5)
N1	0.0277 (4)	0.0321 (4)	0.0230 (3)	−0.0036 (3)	−0.0011 (3)	0.0039 (3)
N2	0.0266 (3)	0.0232 (4)	0.0234 (3)	−0.0059 (3)	0.0027 (3)	0.0005 (3)
C1	0.0223 (4)	0.0226 (4)	0.0272 (4)	−0.0013 (3)	−0.0015 (3)	0.0047 (3)
C2	0.0265 (4)	0.0291 (5)	0.0356 (5)	−0.0040 (3)	0.0025 (3)	0.0091 (4)
C3	0.0253 (4)	0.0243 (4)	0.0534 (6)	−0.0063 (3)	−0.0016 (4)	0.0073 (4)
C4	0.0288 (4)	0.0231 (4)	0.0500 (6)	−0.0040 (3)	−0.0088 (4)	−0.0025 (4)
C5	0.0289 (4)	0.0244 (4)	0.0325 (4)	−0.0022 (3)	−0.0044 (3)	−0.0018 (3)
C6	0.0209 (3)	0.0192 (4)	0.0270 (4)	−0.0013 (3)	−0.0011 (3)	0.0030 (3)
C7	0.0256 (4)	0.0233 (4)	0.0227 (4)	−0.0047 (3)	−0.0037 (3)	0.0036 (3)
C8	0.0279 (4)	0.0222 (4)	0.0281 (4)	−0.0015 (3)	−0.0020 (3)	−0.0005 (3)
C9	0.0264 (4)	0.0314 (4)	0.0232 (4)	−0.0023 (3)	−0.0004 (3)	−0.0013 (3)
C10	0.0537 (7)	0.0509 (7)	0.0340 (5)	−0.0132 (5)	−0.0124 (5)	−0.0054 (5)

### Geometric parameters (Å, °)

**Table:**

O1—C7	1.2349 (10)	C3—H3	0.9500
O1W—H11	0.83 (1)	C4—C5	1.3836 (13)
O1W—H12	0.83 (1)	C4—H4	0.9500
O1W—H13	0.84 (1)	C5—C6	1.4000 (12)
N1—C9	1.2783 (12)	C5—H5	0.9500
N1—C1	1.4108 (12)	C7—C8	1.5086 (12)
N2—C7	1.3484 (11)	C8—C9	1.5081 (12)
N2—C6	1.4088 (10)	C8—H81	0.9900
N2—H2	0.871 (8)	C8—H82	0.9900
C1—C2	1.4038 (12)	C9—C10	1.4937 (14)
C1—C6	1.4061 (12)	C10—H10A	0.9800
C2—C3	1.3769 (15)	C10—H10B	0.9800
C2—H2A	0.9500	C10—H10C	0.9800
C3—C4	1.3894 (16)		
			
H11—O1W—H12	112.1 (16)	C5—C6—C1	119.44 (8)
H11—O1W—H13	110.7 (16)	C5—C6—N2	116.97 (8)
H12—O1W—H13	126 (3)	C1—C6—N2	123.42 (8)
C9—N1—C1	121.45 (8)	O1—C7—N2	122.09 (8)
C7—N2—C6	127.19 (7)	O1—C7—C8	122.73 (8)
C7—N2—H2	116.4 (9)	N2—C7—C8	115.18 (7)
C6—N2—H2	116.0 (9)	C7—C8—C9	108.21 (7)
C2—C1—C6	118.40 (9)	C7—C8—H81	110.1
C2—C1—N1	116.10 (8)	C9—C8—H81	110.1
C6—C1—N1	125.17 (8)	C7—C8—H82	110.1
C3—C2—C1	121.59 (9)	C9—C8—H82	110.1
C3—C2—H2A	119.2	H81—C8—H82	108.4
C1—C2—H2A	119.2	N1—C9—C10	119.67 (9)
C2—C3—C4	119.72 (9)	N1—C9—C8	122.68 (8)
C2—C3—H3	120.1	C10—C9—C8	117.64 (8)
C4—C3—H3	120.1	C9—C10—H10A	109.5
C5—C4—C3	119.93 (9)	C9—C10—H10B	109.5
C5—C4—H4	120.0	H10A—C10—H10B	109.5
C3—C4—H4	120.0	C9—C10—H10C	109.5
C4—C5—C6	120.84 (9)	H10A—C10—H10C	109.5
C4—C5—H5	119.6	H10B—C10—H10C	109.5
C6—C5—H5	119.6		
			
C9—N1—C1—C2	145.61 (9)	N1—C1—C6—N2	4.35 (13)
C9—N1—C1—C6	−41.08 (13)	C7—N2—C6—C5	−147.70 (9)
C6—C1—C2—C3	0.11 (13)	C7—N2—C6—C1	36.97 (13)
N1—C1—C2—C3	173.89 (8)	C6—N2—C7—O1	−178.52 (8)
C1—C2—C3—C4	−2.16 (15)	C6—N2—C7—C8	1.60 (13)
C2—C3—C4—C5	1.76 (14)	O1—C7—C8—C9	112.70 (9)
C3—C4—C5—C6	0.68 (14)	N2—C7—C8—C9	−67.42 (10)
C4—C5—C6—C1	−2.73 (13)	C1—N1—C9—C10	176.32 (9)
C4—C5—C6—N2	−178.25 (8)	C1—N1—C9—C8	−2.65 (13)
C2—C1—C6—C5	2.31 (12)	C7—C8—C9—N1	71.61 (11)
N1—C1—C6—C5	−170.86 (8)	C7—C8—C9—C10	−107.38 (10)
C2—C1—C6—N2	177.52 (7)		

### Hydrogen-bond geometry (Å, °)

**Table:**

*D*—H···*A*	*D*—H	H···*A*	*D*···*A*	*D*—H···*A*
N2—H2···O1^i^	0.87 (1)	2.05 (1)	2.909 (1)	171 (1)
O1w—H11···N1	0.83 (1)	2.12 (1)	2.945 (1)	170 (2)
O1w—H13···O1w^ii^	0.84 (1)	1.98 (1)	2.803 (2)	167 (5)

Symmetry codes: (i) −*x*+1, −*y*+1, −*z*+1; (ii) −*x*+2, −*y*+2, −*z*.
